# Curriculum Innovations: Virtual Didactics as a Tool for Harmonizing Education About Rare Topics in Neuroimmunology

**DOI:** 10.1212/NE9.0000000000200008

**Published:** 2022-09

**Authors:** John Peters, Jeffrey A. Cohen, John R. Corboy, Sarah E. Hopkins, Le H. Hua, Mihir Kakara, Derek McFaul, Ahmed Z. Obeidat, Vijayshree Yadav, Erin E. Longbrake

**Affiliations:** Department of Neurology, Yale School of Medicine, New Haven, CT; Department of Neurology, Mellen MS Center, Neurological Institute, Cleveland Clinic, OH; Rocky Mountain Multiple Sclerosis Center at Anschutz Medical Campus, Universityof Colorado, Denver; Division of Neurology, Children’s Hospital of Philadelphia, University of Pennsylvania; Mellen Program for Multiple Sclerosis, Cleveland Clinic Lou Ruvo Center for Brain Health, Las Vegas, NV; Department of Neurology, University of Pennsylvania Perelman School of Medicine, Philadelphia; Department of Neurology, Oregon Health & Science University, Portland; Department of Neurology, Medical College of Wisconsin, Milwaukee; Department of Neurology, Oregon Health & Science University, Portland; Department of Neurology, Yale School of Medicine, New Haven, CT

## Abstract

**Introduction and Problem Statement:**

Neuroimmunology is a rapidly evolving subspecialty. At this time, fellowship training is not standardized. Discrepancies exist in fellowship programs across the United States, including in faculty expertise in rarer neuroimmunologic conditions. Many graduating fellows feel uncomfortable managing the full spectrum of diseases within neuroimmunology.

**Objectives:**

To evaluate the feasibility and efficacy of a series of live, virtual, interinstitutional seminars educating neuroimmunology fellows on topics that may be infrequently encountered by trainees.

**Methods and Curriculum Description:**

A steering committee of 6 neuroimmunology and multiple sclerosis fellowship program directors selected 18 topics felt to be high yield but representing unique areas of expertise. A live, interactive seminar series was organized. Recognized experts on each topic led seminars using a teleconferencing platform over the 2020–2021 academic year. Recordings were subsequently made available for asynchronous learning. Trainees were surveyed before and after the seminar series and comfort levels with each topic were recorded.

**Results and Assessment Data:**

An average of 41 trainees participated in each live seminar and an additional average of 17 trainees viewed each seminar on demand. Trainee comfort levels with each topic increased after the seminar series was completed. An average of 72% of trainees self-identified as at least “comfortable” with each topic after the series compared with 26% beforehand (*p* < 0.0001).

**Discussion and Lessons Learned:**

A year-long series of live, interactive, interinstitutional seminars focusing on unique topics within a single subspecialty represents an effective way to increase trainee comfort levels with such topics.

Neuroimmunology (NI) is a rapidly evolving subspecialty. New autoimmune diseases of the CNS are recognized and defined every year, and the number of available treatment modalities has increased substantially over the past decade. During a 1- to 2-year fellowship, NI trainees need to develop expertise in an increasing number of rare diseases and immunomodulatory medications.^1,2^ At this time, NI and multiple sclerosis (MS) fellowship training in the United States is not standardized. There is substantial heterogeneity in the duration of training, the size of training programs, the presence of pediatric NI/MS exposure, and the disease entities routinely encountered during training.^3^ In particular, faculty expertise in many of the rarer neuroimmune conditions is unevenly dispersed. Surveys of recent fellowship graduates revealed that even after completing fellowship training, many graduates remained uncomfortable managing the full spectrum of CNS neuroimmune diseases.^4^ Greater standardization of the NI fellowship educational experience is needed. Since it is not possible to ensure homogeneous patient exposures across training programs, improving the quality and range of didactic exposure to rare but important NI topics is paramount.

The coronavirus disease 2019 (COVID-19) pandemic accelerated a shift in medical education toward online learning.^5^ Virtual didactics that represent an opportunity to offer trainees at different institutions synchronized education about rare neuro-immunologic conditions with teaching from international leaders in these areas. This approach harmonizes fellows’ learning and ensures that all trainees receive solid foundational teaching on these topics. We aimed to evaluate the efficacy and feasibility of a nationwide, virtual seminar series educating NI fellows on high-yield topics that may not be routinely encountered.

## Objectives

The program’s objectives were (1) to use a virtual platform to provide national didactics on high-yield, niche topics in NI; (2) to increase comfort levels among NI trainees with such topics; (3) to provide trainees with opportunities for direct interaction with topic experts; and (4) to facilitate discussion and networking among NI trainees. Specific objectives for each seminar were determined by the invited expert faculty leading each seminar.

## Methods and Curriculum Description

### Needs Assessment

A needs assessment was conducted by surveying program directors, trainees, and recent graduates of MS/NI fellowship programs. Surveys were conducted between July 2019 and March 2020.^3,4^ Qualifying individuals were contacted via email and completed surveys using the Qualtrics online survey platform. Recent graduates identified a variety of topics where they felt that additional education during fellowship would have enhanced their transition to independent practice.^4^ Based on these findings, we created a virtual didactic series focusing on (1) niche areas of NI where individual programs would not be universally expected to have high clinical volumes or faculty experts and (2) cross-disciplinary topics identified during the needs assessment as being highly impactful to independent practice.

### Curriculum Development

A core group of 6 educators, all of whom hold subspecialty training in MS/NI, and ad hoc consultants from the leadership of Americas Committee on Treatment and Research in MS (ACTRIMS) was convened for curriculum development. Data from the needs assessment were reviewed, and potential topics/speakers were discussed. Priority was given to topics that would be high yield for trainees, but not covered in individual training programs’ preexisting educational curriculum. These topics aligned with the proposed core curriculum for NI fellowships.^6^ Consensus was reached by majority opinion within the steering committee. Eighteen topics were selected (Table 2). Content validity was verified after the seminar series concluded via survey of program directors on the educational value of each topic. Recognized experts for each topic were invited by the steering committee to lead each seminar.

### Learner Recruitment

The seminar series was advertised to US NI trainees via emails to trainees and program directors. The audience was limited to current or incoming fellows and fellowship directors, with the aim of creating a protected learning environment in which trainees felt comfortable engaging and asking questions. Continuing medical education certification was provided by ACTRIMS. Live biweekly seminars began in August 2020 and continued through June 2021, hosted using the Zoom teleconferencing platform. Recordings were subsequently made available to registered course participants through the ACTRIMS online platform for asynchronous viewing.

### Outcome Measures

Prior to the seminar series, trainees were surveyed on their comfort levels with the individual topics that would be covered by the series. Trainees’ responses were recorded on a 5-point Likert scale ranging from “very uncomfortable” to “very comfortable.” Program directors were also surveyed on the perceived educational value of each topic. Faculty responses were recorded on a 5-point Likert scale ranging from “not very valuable” to “extremely valuable.” Demographic information from both trainees and program directors was also recorded. After the completion of the year-long seminar series, trainees completed the same surveys recording their comfort levels with each topic (Kirkpatrick level 2 assessment). Qualitative feedback on the series was obtained from trainees via open-ended comments. Program directors were surveyed again on the educational value of each topic after the completion of the seminar series.

### Statistical Analysis

Statistical analysis was performed using SPSS version 26 (IBM Corp., Armonk, NY). Weighted averages of trainee comfort level were calculated by assigning values 1 (least comfortable) to 5 (most comfortable) to the 5-point Likert scale responses, summating the responses and averaging by the number of responders. Pretest and posttest weighted averages for each topic were compared using the related samples Wilcoxon signed rank test. Paired pretest and posttest responses were compared using a paired samples *t* test. Differences between first-year and second-year fellows in improvement between pretests and posttests were compared using the Mann-Whitney *U* test.

### Standard Protocol Approvals, Registrations, and Patient Consents

The Yale University Institutional Review Board determined this protocol to be exempt under federal regulation 45 CFR 46.104(d)(4).

### Data Availability

Anonymized data not included in this article may be shared at the request of any qualified investigator for purposes of replicating procedures and results.

## Results and Assessment Data

Seventy-two trainees completed the precourse survey. Table 1 summarizes demographic characteristics of respondents to the precourse and postcourse surveys. In the precourse survey, 18% of trainees identified as residents/incoming fellows, 57% as first-year MS/NI fellows, and 25% as second-year (or beyond) fellows. Across all topics, an average of 74% of trainees felt very uncomfortable, uncomfortable, or neutral with each topic prior to the series. The most frequent response on the Likert scale for each topic was uncomfortable or neutral. Topics that had the lowest comfort level included “hematopoietic and mesenchymal stem cell therapy for MS,” “basic science of remyelination,” “integrative medicine and cannabis,” and “clinical trial design.” The full list of topics is included in Table 2. A total of 122 trainees participated in at least 1 seminar, and 32 trainees participated in at least 50% of the seminars. An average of 41 trainees participated in each live seminar, each of which consisted of a lecture followed by an interactive question and answer period. Attendance during the live seminars waned over the course of the year-long series, with a mean attendance of 53 trainees during the first 5 seminars decreasing to 24 during the final 5 seminars. During the live seminars, trainees actively engaged with presenters, asking an average of 6 comments/questions per seminar with an average of 5 unique commenters per session. A recording of each seminar was made available online after the live session and an additional average of 17 trainees viewed each seminar on-demand. On-demand participation did not wane over the course of the series. The largest proportion of trainees (44%) participated in the live sessions only, with an additional 33% viewing a mixture of live and on-demand seminars, while 22% viewed the seminars on-demand only. These groups did not differ in their geographical make-up.

Twenty-six trainees completed the postcourse survey. An average of 73% of trainees felt either comfortable or very comfortable with each topic after the completion of the series, with the most frequent response being comfortable for every topic (Figure). Weighted averages of trainee comfort level with each topic improved from 2.88 to 3.82 (*p* < 0.001). Weighted averages improved across all topics (Table 2). Matched survey data were available for 14 trainees and showed an increase in mean comfort levels from 2.86 to 3.82 (*p* < 0.001). All 14 trainees demonstrated an improvement in mean comfort levels. Compared with second-year (and beyond) fellows, first-year fellows demonstrated a greater increase in comfort levels (*p* = 0.008). Average comfort level improved from 2.70 to 3.68 among first-year fellows and 3.58 to 4.1 among second-year (and beyond) fellows.

Qualitative feedback from survey participants indicated robust enthusiasm for the seminar series (Table 3). Trainees appreciated the opportunity to interact with leading experts in their fields and commented that the seminars were an improvement from preexisting methods of gaining exposure to these topics. Trainees identified several areas for future growth, including asking facilitators to ensure adequate discussion time and offering ideas for additional topics. Fellowship program directors consistently reported enthusiasm for the topics selected; there was not wide variation in how educationally impactful these topics were felt to be before vs after the seminar series (Table 2).

## Discussion and Lessons Learned

Medical trainees have historically gained exposure to rare disorders and unique topics in a variety of ways, including, among others, independent reading, faculty expertise at their training program, lectures^7^ and simulation cases^8^ within their institution, and online video-based lectures.^9^ Educators attempting to fill gaps in knowledge of such topics have assessed these curricula using tools including self-assessment surveys^10^ and knowledge-based tests.^11^ Despite these efforts, exposure to such niche topics is frequently sporadic and heterogeneous. We report here our experience with implementing a year-long series of live, interactive, interinstitutional seminars focusing on different topics in NI. This educational tool was feasible and helped trainees receive more standardized education on such topics. Precourse/postcourse self-assessments supported the effectiveness of this teaching methodology for increasing trainees’ comfort with the presented material. This expands the repertoire of curricular approaches available to educators, especially in small subspecialties such as NI, in which trainees are expected to develop expertise in a plethora of infrequently encountered topics.

Virtual, interinstitutional approaches have been increasingly used because of the COVID-19 pandemic. These allow for a wide audience across training programs,^12^ can widen the scope of expertise available to trainees,^13,14^ and connect trainees across institutions.^15^ In fellowship programs without a standardized curriculum, such as NI, virtual interinstitutional didactics can help facilitate standardization by providing identical education content across institutions and increasing the accessibility of unequally dispersed expertise. This move toward standardization was not explicitly measured in our study, but qualitative feedback from survey participants indicated that the seminar series improved on previous approaches, such as independent reading.

Several lessons were learned through the development, implementation, and assessment of this seminar series. First, great enthusiasm exists for interactive, interinstitutional learning opportunities among program directors, trainees, and invited faculty. Invited faculty were enthusiastic to participate and having didactics led by a single recognized expert ensures that all trainees have standardized exposure to the core principles related to that topic. The steering committee plays an important role in selecting both high-yield topics and effective educators to present each topic; the latter is particularly important for the success of this curriculum innovation. Live webinars allow for interaction between trainees and experts, and limiting attendance to trainees and program directors ensured that the venue was a safe space for trainees to ask questions. The didactic series was continued for a second iteration during the 2021–2022 academic year and planning is underway for the 2022–2023 series. Based on participant feedback, we continued to offer both live and on-demand options; participants have continued to use both. Several topics were refined or replaced based on participant feedback, and some topics were switched to every-other-year presentations. One area for growth identified by participant feedback focused on leaving enough time for discussion between the experts and the trainees. In response to this, fellow representatives were added to the steering committee, and they served as moderators during the second iteration of the series to ensure that adequate time was reserved for discussion. One of our original aims for this seminar series was to facilitate peer engagement and networking. It was less clear whether this goal was successful, while trainees were exposed to peers’ names and faces during the seminars, question/answer sessions generally did not transition into multiparticipant discussions. Future seminar series will need to consider whether methodological changes might help better achieve this goal.

Barriers to the effectiveness of live webinars included timing; identifying a single time that is reliably convenient for programs across time zones may limit engagement, although this can be mitigated by recording the sessions for asynchronous learning. We observed that trainees in the northeast United States were best represented in our data, which could represent the distribution of training programs (more in the northeast) or the timing of the live seminars. Better outreach to a larger geographical spread of programs could reduce this discrepancy. We found that regularly spacing the series of seminars throughout the academic year allowed for a wide range of topics to be covered, although we also observed a drop-off in attendance toward the end of the year. This might represent conflicting responsibilities or possibly that trainees may have felt they had already mastered the topics under discussion.

The interinstitutional didactic approach is generalizable to other subspecialty fellowships within neurology and across other disciplines, especially those in which exposure to niche topics in unevenly dispersed. It is likely to be best suited for learners focusing on infrequently encountered diagnoses and areas of niche expertise, as common conditions can be well covered by individual institutions.

Trainees received didactic and clinical education from many sources during the year-long course period, so it cannot be assumed that the increase in comfort was solely due to this seminar series. Moreover, a limited number of participants returned the post course survey (26 responses, representing 81% of the 32 most active participants but only 21% of the 122 total course participants). This may bias our data. There were not large variations in attendance between topics, but the number of participants waned gradually over the course of the series. Ways to maintain engagement during future iterations might include offering course certificates for those with sufficient participation or shifting the schedule such that the course occurs biweekly for only 6 months. Follow-up didactic series have yet not incentivized participation.

Overall, trainees found this online seminar series to be an effective and high yield learning opportunity. In a field such as NI, which has rapidly evolving diagnoses and treatments, interinstitutional virtual seminars represent an effective way to help standardize trainee education about rare topics and to enhance overall learning.

## Figures and Tables

**Figure:**
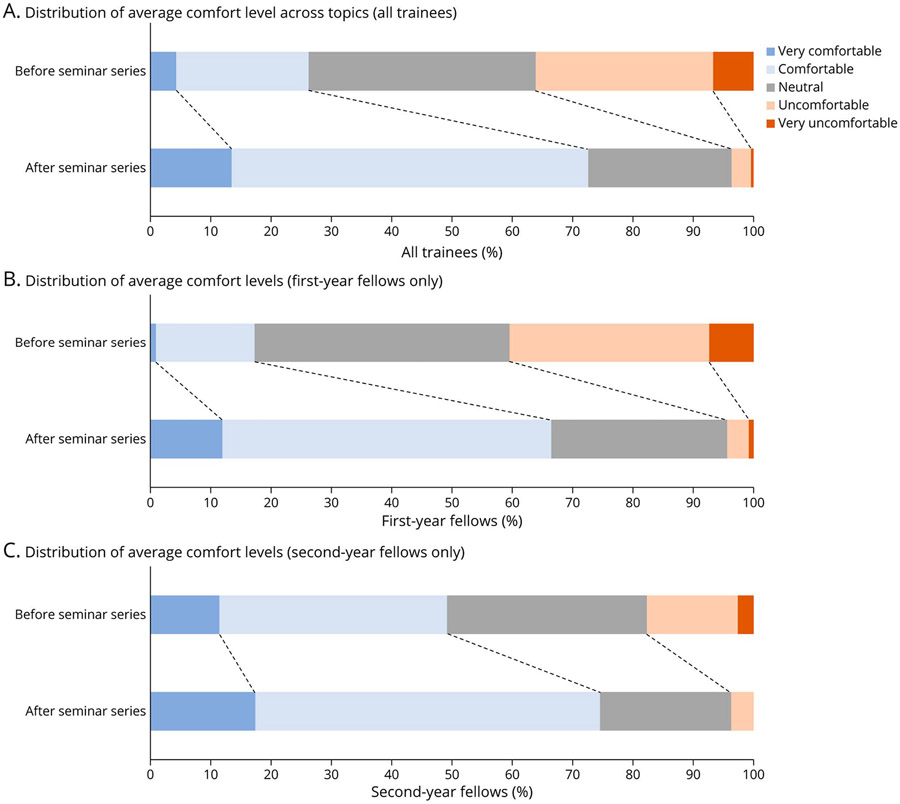
Trainees’ Average Comfort Levels Across All Topics Improved After the Seminar Series: (A) All Trainees, (B) First-Year Fellows Only, and (C) Second-Year (and Beyond) Fellows Only

**Table 1:** Demographic Information

	Percentage of respondents to precourse survey (n = 72)	Percentage of respondents to postcourse survey (n = 26)
**Level of training**		
**Resident**	18.1	11.5
**First-year fellow**	56.9	53.8
**Second-year (or beyond) fellow**	25.0	34.6
**No. of trainees at respondent’s institution**		
**1**	27.1	23.0
**2**	20.3	19.2
**4**	22.0	19.2
**4 or more**	30.5	38.4
**Geographic location of training program**		
**Northeast United States**	39.0	42.3
**Southeast United States**	5.1	3.8
**Midwest United States**	27.1	19.2
**Southwest United States**	11.9	3.8
**West United States**	16.9	30.8

**Table 2:** Weighted Averages Preseries and Postseries for Individual Topics

Topic	Preseries-weighted average of trainee comfort level (n = 72, scale 1–5)	Postseries-weighted average of trainee comfort level (n = 26, scale 1–5)	Preseries-weighted average of program director expected value (n = 22, scale 1–5)	Postseries-weighted average of program director impression of educational value (n = 12, scale 1–5)
**Advanced MRI techniques for MS/neuroinflammation**	2.74	3.77	4.55	4.2
**Anti-NMDA receptor encephalitis and mechanisms of disease**	3.38	4.12	4.59	4.45
**Autoimmune myelitis and its mimics**	3.32	4.12	4.77	4.55
**B-cell and autoantibody-mediated pathogenesis in CNS autoimmunity**	3.09	4.0	4.82	4.36
**Basic science of remyelination**	2.53	3.58	4.36	4.1
**Clinical trial design**	2.55	3.42	4.75	4.27
**Emerging autoimmune diseases**	2.88	3.85	4.73	4.6
**Ethnicity and diversity in the MS experience**	2.92	3.81	4.41	4.45
**Hematopoietic and mesenchymal stem-cell therapy for MS**	2.31	3.54	4.43	4.09
**Immunobiology of MS**	2.93	3.85	4.82	4.44
**Integrative medicine and cannabis**	2.54	3.42	4.18	3.6
**Lab techniques and tools for understanding antibody-mediated diseases**	2.61	3.65	4.5	4.45
**Neurorheumatology: Neurosarcoidosis, CNS vasculitis, CNS lupus, neuro-Beçhet’s**	2.78	3.84	4.82	4.64
**Pediatric MS**	2.74	3.73	4.55	4.4
**PML and opportunistic infections**	3.12	3.96	4.55	4.5
**Progressive MS: challenges in finding and demonstrating effective therapies**	3.2	4.12	4.73	4.42
**Symptomatic management of advanced MS**	3.53	4.27	4.64	3.8
**Urological issues and their management in MS**	2.61	3.73	4.41	4.4

Abbreviations: MS = multiple sclerosis; PML = progressive multifocal leukoencephalopathy.

**Table 3:** Qualitative Feedback From Trainees and Program Directors

Theme	Example quotes
**Benefits of interactions with leading experts**	The lectures are fantastic and it is great to hear from the speakers themselves. Excited to be able to participate myself.
**Improvement on prior approaches for** **learning niche** **and cross-disciplinary topics**	Wonderful seminar series. Love the concept of topics that may not be core topics at each institution or universal.
	Overall, I thought the series was very useful, particularly in covering topics other than MS DMTs.
**Opportunities for live and asynchronous participation**	This was an excellent resource during the MS fellowship, especially the on-demand videos which could be watched at a later time.
**Areas for growth**	Allow more time for discussion—many sessions went over time and it would be great to allow for more engagement.

Abbreviations: DMT = disease-modifying therapy; MS = multiple sclerosis.

## Appendix Authors

**Table:**

Name	Location	Contribution
**John** **Peters, MD**	Department of Neurology, Yale School of Medicine, New Haven, CT	Drafting/revision ofthe manuscript for content, including medical writing for content; major role in the acquisition of data; study concept or design; analysis or interpretation of data
**Jeffrey A. Cohen, MD**	Department of Neurology, Mellen MS Center, Neurological Institute, Cleveland Clinic, OH	Drafting/revision of the manuscript for content, including medical writing for content; study concept or design
**John R. Corboy,** **MD, MA**	Rocky Mountain Multiple Sclerosis Center at Anschutz Medical Campus, University of Colorado, Denver	Drafting/revision of the manuscript for content, including medical writing for content; study concept or design
**Sarah E. Hopkins,** **MS, MSPH**	Division of Neurology, Children’s Hospital of Philadelphia, University of Pennsylvania	Drafting/revision of the manuscript for content, including medical writing for content; study concept or design
**Le H. Hua, MD**	Mellen Program for Multiple Sclerosis, Cleveland Clinic Lou Ruvo Center for Brain Health, Las Vegas, NV	Drafting/revision of the manuscript for content, including medical writing for content; study concept or design
**Mihir** **Kakara,** **MBBS**	Department of Neurology, University of Pennsylvania Perelman School of Medicine, Philadelphia	Drafting/revision of the manuscript for content, including medical writing for content
**Derek McFaul, DO**	Department of Neurology, Oregon Health & Science University, Portland	Drafting/revision of the manuscript for content, including medical writing for content
**Ahmed Z. Obeidat,** **MD, PhD**	Department of Neurology, Medical College of Wisconsin, Milwaukee	Drafting/revision of the manuscript for content, including medical writing for content; study concept or design
**Vijayshree Yadav, MD, MCR**	Department of Neurology, Oregon Health & Science University, Portland	Drafting/revision of the manuscript for content, including medical writing for content; study concept or design
**Erin E.****Longbrake,** **MD, PhD**	Department of Neurology, Yale School of Medicine, New Haven, CT	Drafting/revision of the manuscript for content, including medical writing for content; major role in the acquisition of data; study concept or design; analysis or interpretation of data

## Glossary

ACTRIMS: Americas Committee on Treatment and Research in MS
COVID-19: coronavirus disease 2019
MS: multiple sclerosis
NI: neuroimmunology.
